# Breast Milk Supply of MicroRNA Associated with Leptin and Adiponectin Is Affected by Maternal Overweight/Obesity and Influences Infancy BMI

**DOI:** 10.3390/nu11112589

**Published:** 2019-10-28

**Authors:** Rocío Zamanillo, Juana Sánchez, Francisca Serra, Andreu Palou

**Affiliations:** 1 Laboratory of Molecular Biology, Nutrition and Biotechnology (Group of Nutrigenomics and Obesity), University of the Balearic Islands, 07122 Palma, Spain; oicorzc@gmail.com (R.Z.); joana.sanchez@uib.es (J.S.); andreu.palou@uib.es (A.P.); 2 Department of Health, Valencian International University (VIU), 46002 Valencia, Spain; 3 CIBER de Fisiopatología de la Obesidad y Nutrición (CIBERobn), Instituto de Salud Carlos III (ISCIII), 28029 Madrid, Spain; 4 Instituto de Investigación Sanitaria Illes Balears (IdISBa), 07120 Palma, Spain

**Keywords:** breast milk microRNA, maternal obesity, leptin, adiponectin, infant growth

## Abstract

Breast milk constitutes a dietary source of leptin, adiponectin and microRNAs (miRNAs) for newborns. Expression of miRNAs previously associated with maternal obesity, leptin or adiponectin function were assessed and their impact on infant weight analyzed. Milk samples were collected (at month 1, 2, and 3) from a cohort of 59 healthy lactating mothers (38 normal-weight and 21 overweight/obese (BMI ≥ 25)), and infant growth was followed up to 2 years of age. Thirteen miRNAs, leptin and adiponectin were determined in milk. Leptin, adiponectin and miRNA showed a decrease over time of lactation in normal-weight mothers that was altered in overweight/obesity. Furthermore, negative correlations were observed in normal-weight mothers between the expression of miRNAs in milk and the concentration of leptin or adiponectin, but were absent in overweight/obesity. Moreover, miRNAs negatively correlated with infant BMI only in normal-weight mothers (miR-103, miR-17, miR-181a, miR-222, miR-let7c and miR-146b). Interestingly, target genes of milk miRNAs differently regulated in overweight/obesity could be related to neurodevelopmental processes. In conclusion, a set of miRNAs present in breast milk, in close conjunction with leptin and adiponectin, are natural bioactive compounds with the potential to modulate infant growth and brain development, an interplay that is disturbed in the case of maternal overweight/obesity.

## 1. Introduction

Human milk supplies a number of nutrients and bioactive compounds that drive the development of infants by modulating their metabolic programming and handling genetic, epigenetic and environmental cues, and therefore preparing them for the best outcome in adulthood. Maternal overweight or obesity is deemed to influence the developmental program which could lead to unsuccessful proper growth with consequences in adult life and performance [1]. 

MicroRNAs (miRNAs) are short (21-25 nucleotides) non-coding RNA with the potential to modulate transcription and translation of target miRNA. The recent discovery of miRNAs in breast milk and their high stability has driven attention to their physiological function in lactation [2]. Breast milk constitutes a rich dietary source of miRNAs delivered to new born mammals [3,4]. Although still controversial, milk miRNAs may have the potential of being absorbed by the immature intestinal tract where they may exert their action at a systemic level during early postnatal life [5,6,7,8]. miRNA’s plasma profile has been shown to be altered in gestational [9] and childhood obesity [10,11]. In addition, miRNA’s profile in breast milk may be modulated by both maternal body weight [12] and diet [8,13]. However, little is known regarding the miRNA’s profile in human breast milk and the potential impact on obesity in infants. 

Leptin and adiponectin are two hormones present in maternal milk with a key role in the regulation of energy balance. Leptin has been the focus of intensive research in order to characterize its protective anti-obesity role, particularly during developmental stages [14,15]. Breast milk leptin programs the offspring to be more protected against obesity and other metabolic alterations later on life [16,17,18,19]. Here, we hypothesize that breast milk miRNAs, mostly the ones associated with leptin and adiponectin function, could be altered in overweight/obese lactating mothers and thereby affect growth performance in their infants. Results showed that maternal obesity disturbed the breast milk supply of these miRNAs and their association with milk levels of leptin and adiponectin, as well as their impact on infant BMI at two years of age. 

## 2. Materials and Methods 

### 2.1. Participants and Milk Sample Collection

Fresh milk was obtained from 59 healthy lactating mothers on day 30, 60 and 90 of lactation or as close as possible. Milk was collected (3–12 mL) into sterile recipients between 9 a.m. and 2 p.m. and was kept at 4 °C, transported to the lab the same day and then frozen in working aliquots at −80 °C until further analysis. Milk collection was obtained during the scheduled visits of participants to the midwives. There the researcher involved (R Zamanillo) was present and was responsible for sampling procedure and preservation. To cause minimal disturbances to both mother and the breastfed infant, no specific conditions were imposed on the volunteers for milk sampling. Therefore, milk was collected at the best time of the morning for the mother, normally from both breasts and, usually while the baby was breastfeeding from one side, a milk cup (Medela, Barcelona, Spain) collected the milk from the other breast. In this way, milk collection was performed in a natural and physiological manner and did not cause maternal stress. In addition, a manual breast pump (Medela, Barcelona, Spain) was available during the visit to mothers who preferred this system. In these cases, mothers pumped their breasts after feeding the baby, because it was easier to drain the milk.

Mother–children pairs were enrolled for participation during planned postnatal controls with their midwives. All participants gave written consent. and the protocol was approved by the Ethics Committee of Clinical Research of Balearic Islands (CEIC) (agreement IB 1645/11 PI, July 2011). 

Anthropometric data from mothers and infants were recorded at birth and at the time of sample collection. Follow-up contact was scheduled and performed by phone or e-mail in order to track growth performance of the infants up to 2 years of age. Maternal BMI was calculated as the mean of BMI (body weight self-reported) prior to pregnancy and at months 1, 2 and 3 of lactation. This average BMI was considered to be a good indicator of maternal obesity classification since it reflects perinatal maternal body mass index. 

### 2.2. miRNA Analysis

Firstly, 100 µL of breast milk samples were allowed to thaw in iced water and were homogenized by vortexing for 10 s. Then, small RNAs from breast milk were purified with the microRNA isolation kit mirVana (Kit AM1561, Ambion) following the manufacturer’s instructions and a previously described procedure [2]. The final filtered sample containing small RNAs was eluted in nuclease-free water preheated at 95 °C. The integrity and concentration of RNA was assessed by spectrophotometry using Nanodrop (NanoDrop Technologies, Wilmington, DE, USA) and by capillary electrophoresis using the Small RNA kit (Chip kit 5067-1548) for the Agilent 2100 Bioanalyzer. Quality of RNA preparations was assessed by observing electropherogram profiles; samples showing irregular ones were discarded. 

Reverse transcription (RT) and quantitative PCR (qPCR) of selected miRNAs were performed according to the manufacturer’s protocol. In brief, samples were adjusted to 2.5 ng/µL of small RNAs. Then, 5 µL was reverse-transcribed to cDNA in a 4 µL reaction mix (TaqMan microRNA Reverse Transcription kit, Applied Biosystems) together with 1.5 µL of the miRNA-specific RT primers provided with the TaqMan MicroRNA Assay (Applied Biosystems). RT reactions were performed using an Applied Biosystems 2720 Thermal Cycler (Applied Biosystems) under the following conditions: 16 °C for 30 min; 42 °C for 30 min and 85 °C for 5 min. Then, 2 µL of the miRNA-specific cDNA from RT reaction was amplified with the TaqMan Universal PCR master mix and the respective specific probe provided in the TaqMan MicroRNA Assay (Applied Biosystems). Amplification was initiated at 95 °C for 10 min followed by 40 cycles consisting of denaturation at 95 °C for 15 s, followed by annealing and extension at 60 °C for 1 min. PCR was performed in a StepOnePlusTM Real-Time PCR System (Applied Biosystems). The mature sequence and manufacturer’s reference assay of the selected miRNA are shown in Table S1. 

Selection of miRNAs was based on the literature and the following criteria: they are potential or demonstrated targets for mRNA of leptin (LEP), adiponectin (ADIPOQ), and their respective receptors (LEPR, ADIPOR1 and ADIPOR2); and have been found in fluids (plasma or milk) or associated with obesity [2,4,20]. U6 snRNA was selected as an endogenous control in the comparative cycle threshold method [13]. Expression of hsa-miR-539-5p is tissue specific [21] and was included as a negative control. To ensure reproducibility and to check for day-to-day and plate-to-plate variability, a set of samples were repeated in each RT (two per plate) as internal controls. A maximum of 10% variability between plates was considered acceptable and, on average, the ratio between plates of those samples was 1.001 ± 0.007 (*n* = 18).

In silico target prediction analysis for miRNAs of interest was carried out using miRror 2.0 Suite [22]. Then, a functional enrichment analysis of these targets was conducted by Gene Ontology (GO) annotation and GlueGo+CluePedia Cytoscape plugin [23,24,25].

### 2.3. Analytical Methods 

Milk triglycerides (TG) were determined by commercially available kits (K622-100 from BIOVISION (Milpitas, California)). Individual fatty acids were determined by gas chromatography (GC) in duplicate following the previously described method [26].

Leptin and adiponectin concentration in whole milk samples was determined using commercially available kits. Human Leptin Quantikine ELISA Kit (Quantikine, DLP00) (R&D Systems Europe, Ltd., Abingdon, UK) and ADIPOQ (Human) High Sensitivity ELISA Kit (Abnova, KA0017) (Abnova, Taipei City, Taiwan) were used following the manufacturers´ indications and previously validated protocols in our laboratory [16,27].

### 2.4. Statistical Analysis

Normal distribution of data was tested by Kolmogorov–Smirnov (when *n* ≥ 40) or Shapiro–Wilk (when *n* < 40) tests. When data did not follow a normal distribution, variables were log- or ln- transformed and then considered appropriate. Homogeneity of variances was estimated by Levene’s test. Repeated measures analysis of variance (rANOVA) was used to assess the impact of time (T, month of lactation) and maternal body weight (W, using BMI as a surrogate) on expression of miRNA and analytical variables, and was followed by pairwise comparison to detect differences between months. The associations between miRNA expression and perinatal and maternal variables were tested using Spearman’s correlation test. All statistical analyses were performed using IBM SPSS Statistics 20 and a *p*-value < 0.05 was considered statistically significant. 

## 3. Results

### 3.1. Participant Characteristics

Fifty-nine mothers and their infants completed the study, 64% of whom were normal-weight (BMI < 25) and 36% overweight or obese (BMI ≥ 25). Most of them were of Caucasian origin (90%), had a natural birth (83%), and at term (86%). Almost equal distribution of sexes between infants (53% girls) was observed. At the time of the first milk sampling (1 month post-partum), most of the mothers were following exclusive breastfeeding (86%), which showed a decreasing trend during the subsequent sampling points (85% and 78% at months 2 and 3, respectively). Table S2 shows the characteristics of the whole population, segregated by BMI. 

When considering groups by maternal BMI, no major differences were found regarding maternal and gestational age. A slight tendency towards a higher BMI was found in children from overweight/obese mothers, but this did not attain any statistical significance throughout the period studied (Table S3). 

### 3.2. Expression Levels of miRNAs and Leptin and Adiponectin Concentrations in Breast Milk 

**Figure 1:**
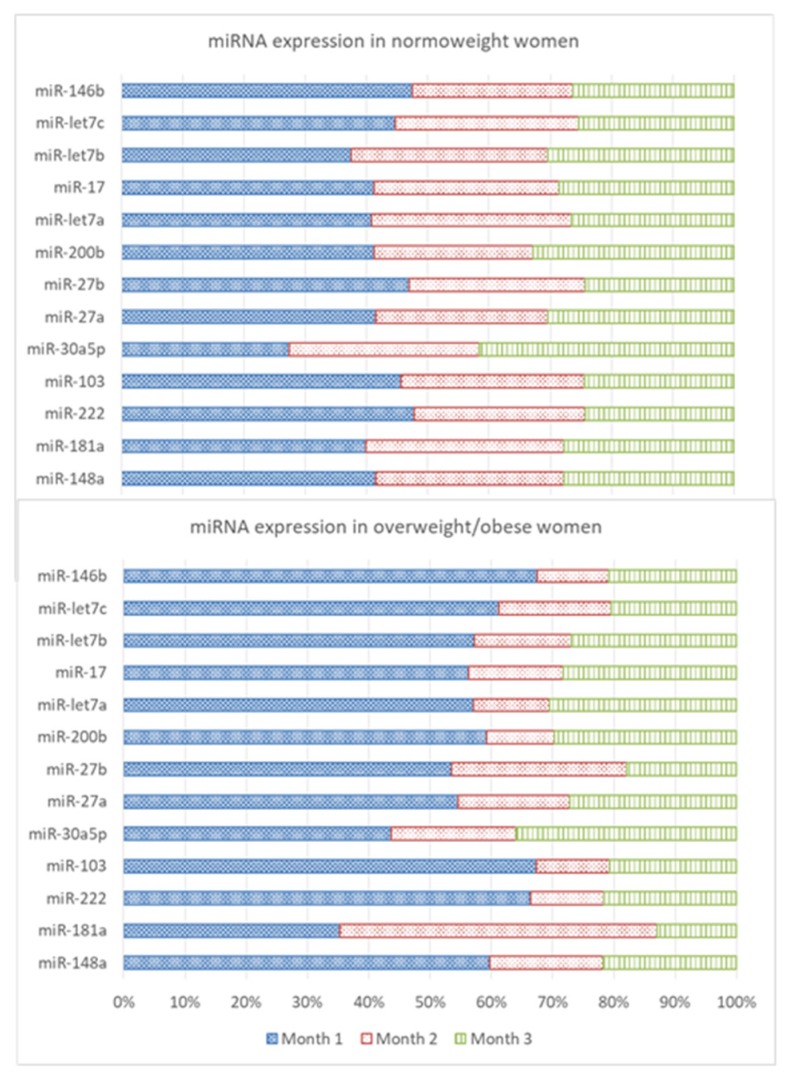
Stacked chart showing the relative percentage of expression of miRNA at month 1, 2, and 3 in normal weight and overweight/obese mothers. The cumulative value of each stacked bar has been defined as 100%.

Thirteen miRNAs, of the twenty-six chosen for this study, were detected in breast milk showing a wide range of activity. miR-30a, miR-146b, miR-let7b, and miR-148a were among the most abundant, whereas miR-27a and miR-27b presented the lowest expression, being around two orders of magnitude lower. Analysis of the whole population showed a decrease in the expression of six miRNAs (miR-222, miR-103, miR-200b, miR-17, miR-let7c, and miR-146b) associated with time of lactation (*p* < 0.05) (Table 1). When maternal BMI was taken into account, a pattern of decreased expression over time, mainly associated with overweight/obesity, was more apparent. Post-hoc analysis of lactating mothers grouped by BMI showed an increase in miR-30a expression in normal-weight mothers (*p* < 0.05) over time that was disturbed in overweight/obese; and a decreased expression of miR-146b that was significant in both groups (*p* < 0.05). The changes observed in the rest of miRNAs showed a general tendency to decrease throughout lactation in normal-weight conditions, but the presence of overweight/obesity unsettled the expression profile. Thus, a smooth decline over time that did not attain statistical significance in normal-weight mothers was observed for the miRNAs analyzed. In contrast, in overweight/obese mothers, expression at month 1 of lactation was much higher but had declined by month 2. This pattern was statistically significant in the overweight/obese population for miR-222, miR-103, miR-17, miR-let7a, and miR-let7c (*p* < 0.05) (Table 1). An overview of the relative percentage of each miRNA the three months studied, shown as 100% stacked bars for each miRNA, allows an easier comparison of miRNA value between normal weight and overweight/obese women (Figure 1).

Milk leptin concentration in the whole population showed a decrease over time of lactation (*p* < 0.05) which was confirmed in normal-weight (*p* < 0.05) but was absent in overweight/obese mothers. Furthermore, overweight/obese mothers showed higher levels of leptin throughout lactation (2.8 higher at month 1) (*p* < 0.05) than normal-weight mothers. Adiponectin concentration was not different between normal-weight and overweight/obese mothers at month 1, but normal-weight mothers showed a decrease over time (≈20%, *p* < 0.05) which was not seen in overweight/obese (Table 2).

### 3.3. In Silico Analysis of Potential miRNA Target Genes 

To further investigate the potential roles of the miRNAs affected by maternal BMI and those that displayed an altered milk level profile over time due to maternal overweight/obesity (miR-let7a, miR-103, miR-222, miR-17, miR-146b, miR-30a), we checked their predicted target genes using miRror 2.0 Suite. 

**Figure 2:**
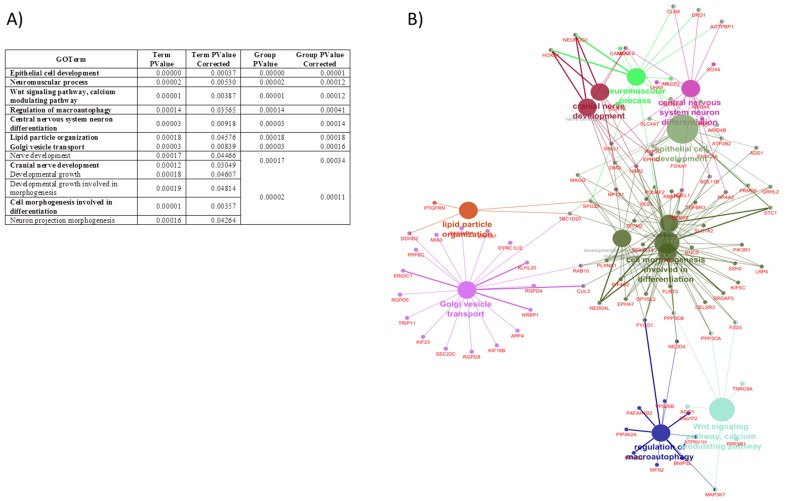
Functional enrichment analysis Gene Ontology (GO) terms for biological process of the predicted target genes of the six of miRNAs which showed altered profiles over time associated with maternal obesity. (**A**) Information on GO terms. *p* values before and after Bonferroni step down correction are indicated. (**B**) Functionally grouped networks with terms as nodes; the most significant terms in the group are labelled in bold. Analysis was conducted using ClueGo + Clupedia app in Cytoscape [23,24,25]

According to miRror, 487 genes are targeted by at least 1 of these miRNAs. All these potential target genes were tested for GO pathway analysis using GlueGo + CluePedia Cytoscape plugin. The genes predicted to be targets of the miRNAs were significantly enriched for 13 term groups that constituted 9 functionally grouped networks: epithelial cell development, neuromuscular process, Wnt signaling pathway and calcium modulating pathway, regulation of macroautophagy, central nervous system neuron differentiation, lipid particle organization, Golgi vesicle transport, cranial nerve development, and cell morphogenesis involved in differentiation (Figure 2). 

### 3.4. Association Analysis of miRNAs with Key Parameters

Correlation analysis was performed with breast milk fatty acid content to assess its potential relationship with miRNAs, as human milk miRNAs have been shown to be influenced by lipid content [5]. 

In normal-weight mothers, negative correlations between miRNAs expression and the monounsaturated fatty acid (MUFA) content in milk were observed at the first month of lactation, which attained statistical significance for miR-222, miR-103, miR-27b, miR-200b, miR-17, and miR-let7b (*p* < 0.05). At month 2, positive correlations were found between milk saturated fatty acid content and miR-181a, miR-103, miR-let7a, miR-let7b, miR-let7c, and miR-17 (*p* < 0.05). Finally, at the third month of lactation, miRNAs expression was positively correlated with milk triglyceride concentration (miR-148a, miR-181a, miR-222, miR-103, miR-30a, miR-200b, miR-17, miR-let7b, miR-let7c, and miR-146b (*p* < 0.05)). Interestingly, this pattern was totally distorted in overweight/obese mothers, in whom statistically significant correlations were only shown at month 2 of lactation; with MUFA positive for miR-103 and miR-200b (*p* < 0.05) and with polyunsaturated fatty acids (PUFA) negative for miR-let7a, miR-let7c, and miR-27a (*p* < 0.05). The whole set of data is included as Table S4.

Furthermore, the association of miRNAs, leptin, and adiponectin levels in milk we analyzed at 2 months of lactation and with infant growth (Table 3). Regarding the correlations of miRNAs and adipokines, results observed in the whole population were mirrored by the normal-weight population but were counteracted by overweight or obese status. Specifically, negative correlations were observed in normal-weight mothers between leptin and miRNAs levels in milk (miR-30a, miR27a, miR-27b, miR-181a, miR-222, miR-103, miR-17, and miR-146b); whereas none of them were found to be either statistically significant or negative in overweight/obese mothers (Table 3). Milk adiponectin negatively correlated with miR-148a, miR-let7a, miR-let7b, miR-103, and miR-17 (*p* < 0.05) in normal-weight mothers; however, no correlations were observed in overweight/obese ones. Interestingly, milk miRNAs were negatively correlated with infant BMI at 24 months of age in normal-weight mothers (miR-103, miR-17, miR-181a, miR-222, miR-let7c, and miR-146b (*p* < 0.05)) whereas no correlations were found in overweight/obese.

## 4. Discussion

We tested the hypothesis that the presence of breast milk miRNAs could be altered in overweight/obese lactating mothers, and there could be a potential relationship with leptin and adiponectin functions in breast milk. Data showed that the supply of miRNAs in maternal milk was altered in overweight/obesity. Furthermore, the associations found between milk miRNAs, leptin, adiponectin, and infant weight were also disturbed in the offspring from overweight or obese mothers. 

The study of miRNAs in breast milk is a topic of great interest in the field of nutrition given their potential involvement in infant development and future health. The relatively immature gastrointestinal tract of newborns may allow effective molecular communication between mother and infant through miRNAs, as suggested by the presence of immune-related miRNAs in mammal milk [2]. Furthermore, growing evidence indicates that miRNAs expressed in breast milk may reflect maternal diet and nutritional status and, therefore, may influence offspring phenotype [8,28].

Human milk is a rich source of lactation-specific miRNAs, which could be used as biomarkers of the performance and health status of both the lactating mammary gland and the breastfed infant [29,30]. Maternal obesity is a factor that may influence miRNA content and expression levels, as has been shown in plasma [9] and placenta [31] during perinatal development. However, the influence of overweight/obesity on the role of miRNAs supplied during breastfeeding is unknown. Here, our approach was to focus on a set of miRNAs with a potential link with obesity and the key regulators leptin and adiponectin, bearing in mind the important role of these proteins in breast milk, particularly leptin, as determinants of a healthy metabolic programming for life as recently revised [15]. Thus, expression and regulation throughout lactation of miRNA genes that are putative targets of leptin (miR-146b, miR-27a, miR-27b, miR-17, miR-30a), adiponectin (miR-181a, miR-27a, miR-27b), and their receptors (miR-let 7a, miR-let7b, miR-let7c, miR-222, miR-30a) or related to adipogenesis and obesity (miR-103, miR-17) were characterized in overweight/obese lactating mothers in comparison with normal-weight mothers. Interestingly, expression of miRNAs associated with obesity was shown (e.g., miR-221) together with others miRNAs previously described as milk-derived miRNAs [32] (e.g., miR-let7a and miR-let7b). In addition, other tissue-specific miRNAs (e.g., miR-95, miR-451) [11,21] could not be detected in breast milk, suggesting a mammary gland-specific delivery of miRNAs to the newborn. 

High variability regarding milk miRNAs content was found between women, as seen in previous studies [2,12]. Data in the normal-weight group were consistent with a smooth decline in the miRNA levels analyzed throughout the first three months of lactation, with the exception of miR-30a, which increased over time. In general terms, our data would fit with the few reports that have analyzed the time course of miRNAs in human breast milk and describe higher levels in early milk, particularly in colostrum, compared with mature milk [2,12]. In contrast, overweight/obese mothers tended to show higher levels during the first month and then sharper decrease in the following months (miR-222, miR-103, miR-17, miR-146b). A number of miRNAs have been linked with obesity or dietary profile [9,33,34], but data on breast milk miRNAs in overweight/obese women as obtained in the present study are lacking. 

Intriguingly, the genes targeted by the six human milk miRNAs that showed altered profiles over time due to overweight/maternal obesity (miR-let7a, miR-103, miR-222, miR-17, miR-146b, miR-30a) were found to be involved in a set of pathways which are very relevant in the developmental process. In this regard, enriched transcription related gene ontology biological processes were connected to three main features: processes involving cell development, morphogenesis and differentiation; a node specifically dealing with neurodevelopment; and another with miRNAs delivery (lipid particle organization and vesicle transport). Involvement of milk miRNAs in infant brain development has already been suggested in preterm delivery [35]. The fact that miRNAs may also be involved in mammary gland signaling during lactation has been shown in dairy cows [36] and humans [37]. All the above points to the fact that milk-miRNAs may influence the growth and development of the newborn, and that maternal overweight/obesity would result in different outcomes from normal-weight mothers. Although we cannot establish a direct cause-effect relationship, it can be hypothesized that in normal weight mothers miRNAs profile in breast milk could modulate metabolic programing in the offspring [38] and this effect can be altered in the case of overweight/obesity. 

Human milk miRNAs are found in breast milk fat globules or enriched in the milk lipid fraction [13,39]. Our analysis was performed in whole milk to ensure total extraction of the miRNAs of interest. To assess the relevance of milk fat on miRNA content, correlation analyses were undertaken. Interestingly, levels of miRNAs in normal-weight mothers were negatively associated with MUFA at the first month of lactation, positively associated with saturated fatty acid content at the second month, and positively associated with triglycerides at the third month. This pattern, absent in overweight/obese women, would suggest a strong dependency of breast milk miRNAs on the quality of the milk fat content, which is influenced by diet [40]. Future studies assessing the impact of dietary fat on breast milk miRNAs and fatty acid content would be necessary to fully understand the secretion of breast milk miRNAs and their regulation. Furthermore, this knowledge could be taken into consideration when designing dietary interventions to optimize breast milk miRNAs in the future. 

Breastfeeding is protective against obesity in adult life [41,42,43], which has been attributed to bioactive compounds that are present in breast milk, but absent in infant formula. Leptin, a main regulator of energy balance, has a key preventive role of obesity during lactation [14,16,44,45,46]. A number of reports have shown a positive correlation between milk leptin and maternal adiposity [47] and a negative association between breast milk leptin, particularly in normal-weight women, and infant weight gain [16,17,46,48]. Evidence recorded in two recent meta-analyses reached the conclusion that breastfeeding is a protective factor against childhood obesity [42,43]. Furthermore, proof that suckling animals supplemented with leptin have a decreased food intake, show healthier food preferences, and are protected against overweight in adulthood, mediated by mechanisms associated with leptin and insulin sensitivity, are critical findings to understand the underlying basis of early human obesity programming [17,19,45,49,50,51]. 

Leptin supply is highly variable, and the main factor conditioning breast milk leptin secretion is the content of maternal body fat stores [47]. Therefore, newborn infants breast fed by obese mothers are exposed to a higher amount of milk leptin; but contrary to expectations, they are more likely to become obese [52], as due to missing the leptin protection role. Further studies are needed to elucidate whether specific milk-derived miRNAs, such as those differentially expressed in overweight/obese in comparison with normal-weight mothers, could be involved in the mechanisms underlying a loss of leptin functionality in the case of obesity.

**Figure 3:**
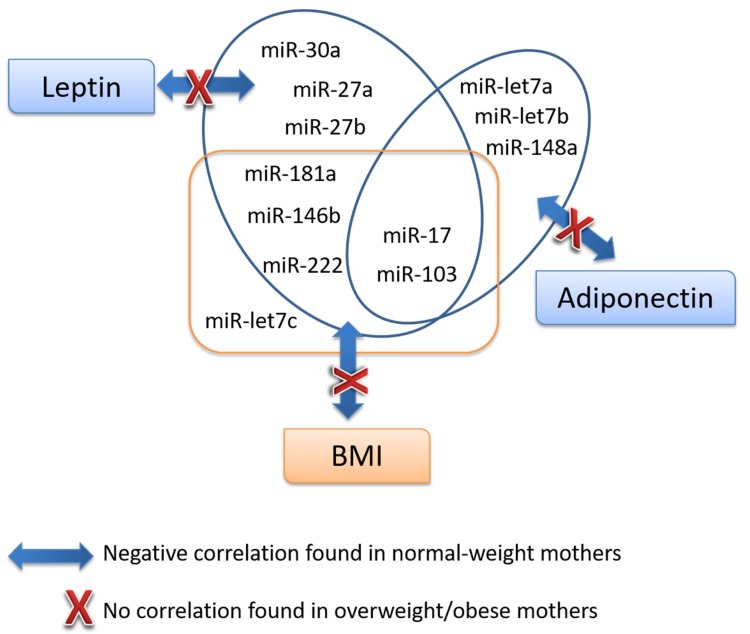
Interactions observed with milk miRNAs, leptin, and adiponectin and their relationship with infant BMI. Schematic representation of the correlations observed between milk miRNAs and leptin, adiponectin, and infant BMI. Milk data are from the second month of lactation and infant BMI corresponds to data collected at 24 months of age. These relationships were observed in normal-weight mothers but were absent in overweight/obesity.

In contrast with leptin, the role of adiponectin affecting metabolic regulation and programming of developing infants is less known, but it has been described that higher milk levels could be associated with greater adiposity in offspring [44]. In this context, our data give shape to the idea that a set of miRNAs present in breast milk may act, in close conjunction with leptin and adiponectin, as bioactive compounds with the potential to modulate infant body weight and composition (Figure 3). Potentially, common modulatory mechanisms of secretion in the mammary gland could be responsible for this association, which remains to be tested. Our data are suggestive of an interplay between leptin, adiponectin and a core of miRNAs in milk, which in the end modulate the BMI of infants at 2 years of age. Furthermore, it is apparent that maternal overweight/obesity alters this interplay. To gain a better understanding of the mechanism of action would enable better strategies in the prevention of obesity, including better nutrition of mothers to optimize milk composition and, when breastfeeding is not possible, this knowledge would potentially contribute to the improvement of infant formula to support optimized growth. 

## Figures and Tables

**Table 1:** MicroRNAs (miRNAs) in breast milk during lactation.

		Month 1					Month 2					Month 3					Statistical Analysis	
		N	Min	Max	Mean	SD	N	Min	Max	Mean	SD	N	Min	Max	Mean	SD	Overall	Normal/Obese Weight
miR-148a	Whole	52	0.004	237.21	21.98	45.40	53	0.061	69.52	11.28	13.73	55	0.029	88.37	11.13	15.46		
	Normal-weight	34	0.004	124.38	15.77	24.35	36	0.063	69.52	11.69	14.62	36	0.262	62.59	10.54	12.13		
	Overweight/obese	18	0.018	237.21	33.71	69.36	17	0.061	42.12	10.41	12.02	19	0.029	88.37	12.24	20.72		
miR-181a	Whole	53	0.024	34.72	2.93	5.36	54	0.060	53.90	3.33	10.11	55	0.041	14.48	1.49	2.22		
	Normal-weight	34	0.024	10.14	1.99	2.14	36	0.060	7.97	1.61	1.94	36	0.067	7.64	1.38	1.41		
	Overweight/obese	19	0.115	34.72	4.63	8.36	18	0.068	53.90	6.78	17.09	19	0.041	14.48	1.69	3.29		
miR-222	Whole	51	0.017	31.63	2.99 ^a^	5.89	53	0.032	6.46	1.04 ^a,b^	1.14	54	0.024	9.75	1.20 ^b^	1.63	T (*p* = 0.020)	
	Normal-weight	34	0.017	12.48	1.88	2.43	35	0.065	6.46	1.10	1.20	36	0.056	2.46	0.96	0.73	T (*p* = 0.006)	
	Overweight/obese	17	0.108	31.63	5.22 ^a^	9.40	18	0.032	4.09	0.94 ^ab^	1.05	18	0.024	9.75	1.70 ^b^	2.60		T (*p* = 0.029)
miR-103	Whole	52	0.019	21.09	2.21 ^a^	3.81	54	0.036	7.05	0.90 ^b^	1.25	54	0.013	7.70	0.91 ^b^	1.28	T (*p* = 0.019)	
	Normal-weight	34	0.019	11.80	1.60	2.27	36	0.036	7.05	1.05	1.47	36	0.053	4.52	0.85	0.91	TxW (*p* = 0.022)	
	Overweight/obese	18	0.105	21.09	3.36 ^a^	5.61	18	0.070	2.02	0.59 ^b^	0.52	18	0.013	7.70	1.03 ^b^	1.83		T (*p* = 0.017)
miR-30a	Whole	51	0.474	410.65	47.84	71.53	53	0.355	220.17	37.26	41.28	55	1.66	510.41	54.88	78.87		
	Normal-weight	33	0.474	112.54	34.48 ^a^	34.18	35	1.64	220.17	39.04 ^b^	44.04	36	2.85	252.94	52.31 ^b^	51.76	TxW (*p* = 0.023)	T (*p* = 0.008)
	Overweight/obese	18	1.70	410.65	72.33	108.94	18	0.355	128.04	33.81	36.27	19	1.66	510.41	59.75	115.82		
miR-27a	Whole	52	0.004	4.68	0.40	0.81	54	0.008	1.84	0.18	0.27	55	0.008	4.09	0.23	0.56		
	Normal-weight	34	0.004	1.56	0.24	0.29	36	0.009	0.67	0.16	0.15	36	0.016	0.91	0.17	0.21	T (*p* = 0.049)	
	Overweight/obese	18	0.056	4.68	0.70	1.29	18	0.008	1.84	0.23	0.42	19	0.008	4.09	0.35	0.92		
miR-27b	Whole	52	0.003	1.44	0.19	0.27	54	0.002	1.50	0.11	0.21	52	0.002	0.65	0.08	0.11		
	Normal-weight	34	0.003	0.89	0.15	0.18	36	0.002	0.57	0.09	0.11	35	0.003	0.40	0.08	0.08	T (*p* = 0.022)	
	Overweight/obese	18	0.007	1.44	0.26	0.39	18	0.004	1.50	0.14	0.34	17	0.002	0.65	0.09	0.16		
miR-200b	Whole	52	0.041	78.38	9.06 ^a^	14.69	49	0.002	24.40	3.68 ^b^	4.48	53	0.066	40.98	5.87 ^ab^	7.71	T (*p* = 0.026)	
	Normal-weight	34	0.088	36.53	6.87	8.35	32	0.007	24.40	4.33	5.17	34	0.138	23.98	5.46	5.12		
	Overweight/obese	18	0.041	78.38	13.20	22.01	17	0.002	8.00	2.46	2.46	19	0.066	40.98	6.60	11.07		
miR-let7a	Whole	51	0.063	56.35	5.44	10.03	48	0.058	18.65	2.64	3.74	51	0.050	26.57	3.25	4.56		
	Normal-weight	34	0.063	29.79	3.85	5.78	31	0.058	18.65	3.06	4.33	33	0.050	8.91	2.50	2.01	TxW (*p* = 0.045)	
	Overweight/obese	17	0.200	56.35	8.62 ^a^	15.14	17	0.063	8.38	1.87 ^b^	2.24	18	0.079	26.57	4.62 ^ab^	7.11		
miR-17	Whole	52	0.047	34.02	4.88 ^a^	6.39	52	0.083	17.68	2.55 ^b^	3.31	55	0.069	17.34	2.95 ^b^	3.42	T (*p* = 0.029)	
	Normal-weight	34	0.047	21.32	4.01	4.50	34	0.083	17.68	2.94	3.79	36	0.126	13.37	2.78	2.74	TxW (*p* = 0.034)	
	Overweight/obese	18	0.144	34.02	6.52 ^a^	8.89	18	0.180	6.48	1.80 ^b^	2.01	19	0.069	17.34	3.27 ^ab^	4.50		T (*p* = 0.048)
miR-let7b	Whole	52	0.157	244.25	26.50	41.50	54	0.316	85.16	14.79	17.44	55	0.584	89.15	16.86	19.25		
	Normal-weight	34	0.157	93.52	19.64	19.91	36	0.316	85.16	16.77	19.66	36	0.584	89.15	15.94	16.30		
	Overweight/obese	18	0.659	244.25	39.47	64.23	18	0.773	39.07	10.83	11.30	19	0.714	83.21	18.60	24.30		
miR-let7c	Whole	51	0.035	23.88	2.88 ^a^	4.54	54	0.024	9.21	1.37 ^ab^	1.76	55	0.053	7.04	1.27 ^b^	1.48	T (*p* = 0.014)	
	Normal-weight	33	0.035	9.31	2.10	1.97	36	0.024	6.70	1.41	1.62	36	0.053	5.85	1.19	1.12	T (*p* = 0.016)	
	Overweight/obese	18	0.079	23.88	4.32 ^a^	7.07	18	0.067	9.21	1.30 ^a,b^	2.07	19	0.057	7.04	1.44 ^b^	2.03		
miR-146b	Whole	50	0.148	354.36	35.70 ^a^	65.49	48	0.427	62.18	12.02 ^b^	12.85	53	0.034	78.81	14.77 ^b^	17.66	T (*p* = 0.008)	
	Normal-weight	32	0.148	103.03	24.10 ^a^	29.06	31	0.448	62.18	13.26 ^b^	13.15	34	0.034	44.90	13.32 ^b^	12.60	T (*p* = 0.001)	T (*p* = 0.029)
	Overweight/obese	18	0.890	354.36	56.31 ^a^	100.61	17	0.427	40.83	9.75 ^b^	12.33	19	0.090	78.81	17.37 ^b^	24.48		T (*p* = 0.027)

Breast milk miRNAs were purified with the microRNA isolation kit mirVana and determined in breast milk at month 1, 2, and 3 of lactation by TaqMan microRNA specific assays. Whole population was segregated at sampling time into normal-weight (BMI < 25) and overweight/obese (BMI ≥ 25) mothers. Repeated measures analysis of variance (rANOVA) was used to assess the impact of time of lactation (T) on the whole population, and then to explore the influence of maternal overweight/obesity (W) throughout lactation (T). This was followed by pairwise post-hoc comparison to detect differences between months. Statistically significant differences were considered when *p* < 0.05, and are indicated in the columns overall and normal/obese-weight; in addition, significances that appeared by pair comparison are represented in data not sharing the same superscript (in the same row). N (number of subjects); Min (minimum); Max (maximum); SD (standard deviation). The miRNAs studied are described as miR- followed by their specific code.

**Table 2:** Breast milk leptin and adiponectin.

		Month 1					Month 2					Month 3					Statistical Analysis	
		N	Min	Max	Mean	SD	N	Min	Max	Mean	SD	N	Min	Max	Mean	SD	Overall	Normal/Obese Weight
Leptin (ng/mL)	Whole	53	0.020	1.92	0.376 ^a^	0.353	55	0.003	1.18	0.341 ^b^	0.314	54	0.003	1.45	0.346 ^b^	0.333	Normal/Obese weight	T (*p* = 0.010)
	Normal-weight	35	0.020	0.60	0.235 ^a^	0.153	36	0.003	0.98	0.242 ^b^	0.242	35	0.020	1.08	0.209 ^b^	0.195	T (*p* = 0.030); W (*p* = 0.000)	
	Overweight/obese	18	0.156	1.92	0.652	0.462	19	0.020	1.18	0.529	0.355	19	0.003	1.45	0.597	0.388		
Adiponectin (ng/mL)	Whole	53	8.90	48.50	23.39	7.57	55	7.30	44.10	20.83	6.61	53	9.30	65.70	20.32	8.02		T (*p* = 0.000)
	Normal-weight	35	10.70	48.50	23.16 ^a^	7.97	36	7.30	44.10	19.19 ^b^	7.03	35	9.30	26.80	18.81 ^b^	4.62	TxW (*p* = 0.035)	
	Overweight/obese	18	8.90	34.00	23.03	6.94	19	10.00	31.50	22.37	5.58	18	13.30	65.70	23.38	11.80		

Breast milk leptin and adiponectin were determined by ELISA at month 1, 2, and 3 of lactation. Whole population was segregated at sampling time into normal-weight (BMI < 25) and overweight/obese (BMI ≥ 25) mothers. Repeated measures analysis of variance (rANOVA) was used to assess the impact of time of lactation (T) on the whole population, and then to explore the influence of maternal overweight/obesity (W) throughout lactation (T), which was followed by pairwise post-hoc comparison to detect differences between months. Statistically significant differences were considered when *p* < 0.05 and are indicated in the respective columns of overall and normal/obese-weight; in addition, significances that appeared by pair comparison are represented in data not sharing the same superscript (in the same row). N (number of subjects); Min (minimum); Max (maximum); SD (standard deviation).

**Table 3:** Correlations between milk leptin, adiponectin and miRNAs at the second month of lactation and their relationship with infant BMI at 2 years of age.

			Adiponectin	miR-30a	miR-27a	miR-27b	miR-148a	miR-let7a	miR-let7b	miR-103	miR-17	miR-181a	miR-222	miR-let7c	miR-146b	miR-200b
**Leptin**	Whole	r	0.214	−0.195	−0.092	−0.165	−0.064	−0.146	−0.151	−0.325 ^*^	−0.198	−0.172	−0.162	−0.110	−0.263	−0.184
		p	0.117	0.161	0.508	0.233	0.647	0.322	0.275	0.016	0.160	0.214	0.246	0.428	0.071	0.206
	Normal-weight	r	0.089	−0.454 ^**^	−0.380 ^*^	−0.410 ^*^	−0.150	−0.328	−0.276	−0.574 ^**^	−0.419 ^*^	−0.470 ^**^	−0.471 ^**^	−0.261	−0.473 ^**^	−0.297
		p	0.605	0.006	0.022	0.013	0.381	0.072	0.103	0.000	0.014	0.004	0.004	0.125	0.007	0.098
	Overweight/Obese	r	0.136	0.159	0.352	0.180	0.112	0.170	0.248	0.114	0.301	0.267	0.386	0.214	0.139	0.055
		p	0.580	0.529	0.152	0.476	0.670	0.513	0.321	0.654	0.224	0.283	0.113	0.394	0.596	0.833
Adiponectin	Whole	r	1,000	−0.345 ^*^	−0.123	−0.198	−0.353 ^**^	−0.374 ^**^	−0.403 ^**^	−0.365 ^**^	−0.425 ^**^	−0.275 ^*^	−0.310 ^*^	−0.329^*^	−0.293 ^*^	−0.248
		p		0.011	0.374	0.152	0.010	0.009	0.003	0.007	0.002	0.044	0.024	0.015	0.043	0.086
	Normal-weight	r	1,000	−0.325	−0.071	−0.134	−0.388 ^*^	−0.467 ^**^	−0.384 ^*^	−0.393^*^	−0.418 ^*^	−0.204	−0.299	−0.319	−0.240	−0.207
		p		0.057	0.682	0.435	0.019	0.008	0.021	0.018	0.014	0.232	0.081	0.058	0.193	0.255
	Overweight/Obese	r	1,000	−0.209	−0.184	−0.291	−0.222	−0.006	−0.151	−0.180	−0.250	−0.164	−0.163	−0.313	−0.085	−0.098
		p		0.406	0.465	0.241	0.392	0.981	0.550	0.476	0.317	0.515	0.518	0.206	0.747	0.708
BMI24	Whole	r	0.125	−0.246	−0.167	−0.218	−0.252	−0.326 ^*^	−0.278	−0.425 ^**^	−0.298 ^*^	−0.238	−0.298 ^*^	−0.331 ^*^	−0.287	−0.347 ^*^
		p	0.380	0.085	0.245	0.129	0.081	0.031	0.051	0.002	0.040	0.096	0.037	0.019	0.059	0.020
	Normal-weight	r	0.199	−0.302	−0.196	−0.276	−0.207	−0.296	−0.313	−0.521 ^**^	−0.427 ^*^	−0.347 ^*^	−0.385 ^*^	−0.360 ^*^	−0.422 ^*^	−0.327
		p	0.260	0.082	0.267	0.114	0.239	0.120	0.071	0.002	0.015	0.044	0.027	0.036	0.023	0.078
	Overweight/Obese	r	−0.124	−0.224	−0.179	−0.071	−0.377	−0.379	−0.285	−0.265	−0.171	−0.106	−0.138	−0.276	−0.218	−0.318
		p	0.636	0.405	0.506	0.795	0.166	0.164	0.284	0.322	0.528	0.696	0.610	0.300	0.435	0.248

Whole population was segregated at sampling time into normal-weight mothers (BMI < 25) and overweight/obese (BMI ≥ 25). Associations between milk leptin, adiponectin, and miRNAs in breast milk at the second month of lactation and with infant BMI at 24 months of age (BMI24) were tested by Spearman’s correlation test. Spearman's rank correlation coefficient (r) and significance (*p*) are shown. ** = *p* < 0.01 (bilateral); * = *p* < 0.05 (bilateral). The miRNAs studied are described as miR- followed by their specific code.

## Supplementary Materials

The following are available online at https://www.mdpi.com/2072-6643/11/11/2589/s1, Table S1. miRNAs selected to assay in breast milk from lactating mothers. Table S2. Participant characteristics. Table S3. Maternal data and infant anthropometry. Table S4. Correlations between miRNAs during lactation and fatty acid content in milk.
