# Supplementary material for: Breast Milk Supply of MicroRNA Associated with Leptin and Adiponectin Is Affected by Maternal Overweight/Obesity and Influences Infancy BMI
Source: Nutrients. 2019 Oct 28;11(11):2589. doi: 10.3390/nu11112589 (PMC6893542; doi:10.3390/nu11112589)
Supplement: Supplementary file 1 [file nutrients-11-02589-s001.pdf]

Breast milk supply of microRNA associated with leptin and adiponectin is affected by maternal overweight/obesity and influences infancy BMI

Rocío Zamanillo[1,4], Juana Sánchez[1,2,3,5], Francisca Serra[1,2,3,6]* and Andreu Palou[1,2,3,7]

1 Laboratory of Molecular Biology, Nutrition and Biotechnology (Group of Nutrigenomics and Obesity), University of the Balearic Islands 07122 Palma, Spain
2 CIBER de Fisiopatología de la Obesidad y Nutrición (CIBERobn), Instituto de Salud Carlos III (ISCIII), Madrid, Spain
3 Instituto de Investigación Sanitaria Illes Balears (IdISBa).

* Correspondence: francisca.serra@uib.es. Tel.: +34 971173051

Table S1. miRNA selected to assay in breast milk from lactating mothers.

miRNA	Mature sequence	Reference assay[1]	Main criteria for selection
hsa-let-7a-5p	UGAGGUAGUAGGUUGUAUAGUU	000377	Target of ADIPOR2, LEPR<br>Found in human breast milk
hsa-let-7b-5p	UGAGGUAGUAGGUUGUGUGGUU	000378	Target of ADIPOQ, ADIPOR2, LEPR<br>Found in human breast milk
hsa-let-7c-5p	UGAGGUAGUAGGUUGUAUGGUU	000379	Target of ADIPOQ, ADIPOR2, LEPR
hsa-miR-103a-3p	AGCAGCAUUGUACAGGGCUAUGA	000439	Found in human breast milk
hsa-miR-122a	UGGAGUGUGACAAUGGUGUUUGU	000445*	Liver-specific and found in cow milk
hsa-miR-126-3p	UCGUACCGUGAGUAAUAAUGCG	002228*	Involved in obesity
hsa-miR-143-3p	UGAGAUGAAGCACUGUAGCUCA	000466†	Target of ADIPOQ<br>Involved in obesity
hsa-miR-145-5p	GUCCAGUUUUCCCAGGAAUCCCU	002278*	Involved in obesity
hsa-miR-146b-5p	UGAGAACUGAAUUCCAUAGGCU	001097	Target of LEP<br>Found in human breast milk
hsa-miR-148a-3p	UCAGUGCACUACAGAACUUUGU	000470	Highly abundant in human breast milk
hsa-miR-150-5p	UCUCCCAACCCUUGUACCAGUG	000473*	Target of LEPR, ADIPOR2
hsa-miR-153-3p	UUGCAUAGUCACAAAAGUGAUC	001191*	Target of ADIPOQ
hsa-miR-17-5p	CAAAGUGCUUACAGUGCAGGUAG	002308	Target of LEP, ADIPOR2<br>Found in plasma and involved in obesity

hsa-miR-181a-5p	AACAUUCAACGCUGUCGGUGAGU	000480	Target of ADIPOQ, LEPR<br>Found in breast milk and involved in obesity
hsa-miR-200b-3p	UAAUACUGCCUGGUAAUGAUGA	002251	Target of ADIPOQ, ADIPOR2, LEPR
hsa-miR-221-3p	AGCUACAUUGUCUGCUGGGUUUC	000524*	Target of ADIPOR1<br>Found in plasma and involved in obesity
hsa-miR-222-3p	AGCUACAUCUGGCUACUGGGU	002276	Target of ADIPOR1<br>Found in plasma and involved in obesity
hsa-miR-26a-5p	UUCAAGUAAUCCAGGAUAGGCU	000405*	Target of ADIPOR1<br>Found in human breast milk
hsa-miR-26b-5p	UUCAAGUAAUUCAGGAUAGGU	000407†	Target of ADIPOR1
hsa-miR-27a-3p	UUCACAGUGGCUAAGUUCCGC	000408	Target of LEP, ADIPOQ<br>Involved in obesity
hsa-miR-27b-3p	UUCACAGUGGCUAAGUUCUGC	000409	Target of LEP, ADIPOQ
hsa-miR-30a-5p	UGUAAACAUCCUCGACUGGAAG	000417	Target of LEP, LEPR
hsa-miR-384	AUUCCUAGAAAUUGUUCAUA	000574*	Target of LEPR<br>Associated with adipogenesis
hsa-miR-451	AAACCGUUACCAUUACUGAGUUU	001105*	Target of ADIPOQ
hsa-miR-539-5p	GGAGAAAUUAUCCUUGGUGUGU	001286*	Expressed in human HEK-239 cells
hsa-miR-95-3p	UUCAACGGGUAUUUAUUGAGCA	000433*	Expressed in adipose tissue
U6 snRNA	GTGCTCGCTTCGGCAGCACATATACT<br>AAAATTGGAACGATACAGAGAAGATT<br>AGCATGGCCCCTGCGCAAGGATGACAC<br>GCAAATTCGTGAAGCGTTCCATATTTT	001973	Control

1: Data obtained from www.thermofisher.com, accessed on 07/03/2016

* miRNA were undetected or undetermined in the milk samples.

† Data showed very high variability between samples or low efficiency at amplification and were not considered for analysis.

Selection of miRNA was based on their role as targets for mRNA of leptin (LEP), adiponectin (ADIPOQ) or their respective receptors (LEPR, ADIPOR1 and ADIPOR2), described in fluids (plasma or milk) or associated with obesity. U6 snRNA was selected as the endogenous control in the comparative cycle threshold method.

Table S2. Participant characteristics

			Whole population (n=59)	Normal-weight (n=38)	Overweight/ obese (n=21)
Sex	Boy/Girl		28/31	16/22	12/9
Location	Capital/ Neighborhood/ Town		21/13/25	12/9/17	9/4/8
Ethnicity	Caucasian/Other		53/6	35/3	18/3
Delivery	Natural/Caesarean		49/10	35/3	14/7
	Winter/Summer		38/21	25/13	13/8
	Term/Pre-term/Post-term		51/5/3	35/2/1	16/3/2
Lactation	Breastfeeding/mixed	Month 1	51/8	36/2	15/6
		Month 2	50/9	34/4	16/5
		Month 3	46/13	32/6	14/7

Characteristics of the cohort of lactating mothers recruited for participating in the present study. The whole population was segregated in normal-weight mothers (BMI<25) and overweight or obese (BMI≥25).

Table S3. Maternal data and infant anthropometry

		Whole population					Normal-weight					Overweight/Obese				
		N	Min	Max	Mean	SD	N	Min	Max	Mean	SD	N	Min	Max	Mean	SD
**Mother**																
Age		59	25.0	42.0	32.0	3.48	38	25.0	42.0	32.2	3.42	21	25.0	41.0	31.6	3.65
BMI (kg/m^2)		59	17.46	38.30	24.07	4.44	38	17.46	24.57	21.43	1.85	21	25.1	38.30	28.87	3.66
Gestational age (weeks)		59	34.0	42.0	39.1	1.73	38	34.0	42.0	39.2	1.56	21	34.0	42.0	38.8	2.02
**Infant**																
Body weight (kg)	at birth	59	1.87	4.17	3.19	0.52	38	2.06	4.17	3.23	0.44	21	1.87	4.06	3.11	0.64
	Month 1	59	2.60	5.86	4.00	0.71	38	3.00	5.86	3.94	0.68	21	2.60	5.55	4.11	0.78
	Month 2	59	3.50	7.12	5.04	0.82	38	3.50	7.12	4.98	0.81	21	3.74	6.88	5.14	0.86
	Month 3	59	4.08	8.01	5.88	0.84	38	4.55	8.01	5.80	0.77	21	4.08	7.69	6.03	0.96
	Month 6	59	5.89	9.85	7.51	0.91	38	5.89	9.85	7.44	0.86	21	5.91	9.49	7.66	1.00
	Month 12	59	6.98	12.47	9.42	1.15	38	6.98	12.39	9.31	1.05	21	7.47	12.47	9.63	1.32
	Month 18	59	8.24	14.60	10.86	1.23	38	8.24	14.60	10.71	1.18	21	9.30	13.89	11.13	1.28
	Month 24	54	9.50	16.70	12.35	1.45	35	9.50	16.70	12.24	1.42	19	10.38	15.32	12.55	1.53
Length (cm)	at birth	58	43.00	53.00	49.30	2.15	37	46.00	53.00	49.30	1.71	21	43.00	53.00	49.31	2.80
	Month 1	59	46.50	58.50	52.99	2.90	38	46.50	58.00	52.83	2.68	21	46.50	58.50	53.26	3.31
	Month 2	59	51.00	63.00	56.53	2.93	38	51.00	63.00	56.43	2.85	21	51.00	62.00	56.71	3.14
	Month 3	59	53.00	66.50	59.87	3.06	38	53.00	66.50	59.76	2.97	21	53.00	64.50	60.07	3.30
	Month 6	59	60.00	73.00	66.75	2.74	38	61.50	73.00	66.68	2.75	21	60.00	71.50	66.88	2.78
	Month 12	59	68.00	82.00	75.19	2.97	38	68.00	82.00	75.09	2.95	21	70.00	81.00	75.38	3.07
	Month 18	59	75.50	89.00	81.83	3.00	38	75.50	87.50	81.49	2.79	21	76.00	89.00	82.45	3.34
	Month 24	54	79.00	95.00	87.64	3.11	35	79.00	94.00	87.44	3.13	19	81.50	95.00	88.03	3.12
BMI (kg/m^2)	BMI1	59	11.04	17.42	14.14	1.42	38	11.04	17.42	14.03	1.44	21	11.88	16.74	14.33	1.39
	BMI2	59	12.76	20.11	15.69	1.53	38	12.76	20.11	15.57	1.57	21	13.82	19.10	15.89	1.47

	BMI3	59	13.30	19.77	16.35	1.37	38	13.30	19.77	16.20	1.30	21	14.33	19.35	16.61	1.49
	BMI6	59	14.37	19.75	16.82	1.33	38	14.84	19.75	16.69	1.24	21	14.37	19.56	17.06	1.49
	BMI12	59	14.48	19.98	16.63	1.34	38	14.48	19.27	16.48	1.19	21	14.68	19.98	16.89	1.56
	BMI18	59	13.63	19.07	16.18	1.13	38	13.63	19.07	16.10	1.16	21	14.14	18.78	16.33	1.07
	BMI24	54	13.87	19.76	16.04	1.29	35	13.87	18.90	15.97	1.25	19	14.09	19.76	16.16	1.40

Whole population was segregated in normal-weight mothers (BMI<25) and overweight or obese (BMI≥25) at sampling time. Infant data was recorded from birth up to 2 years old.

Analysis of variance (ANOVA) was used to assess the impact of maternal obesity on the data collected. No statistical significant differences ($p<0.05$) were found.

Table S4. Correlations between miRNA during lactation and fatty acid content in milk.

		Normal-weight												Overweight/obese											
		Month 1				Month 2				Month 3				Month 1				Month 2				Month 3			
		SFA	MUFA	PUFA	TG	SFA	MUFA	PUFA	TG	SFA	MUFA	PUFA	TG	SFA	MUFA	PUFA	TG	SFA	MUFA	PUFA	TG	SFA	MUFA	PUFA	TG
miR-148a	r	-.088	-.272	.264	.156	.096	-.029	-.045	.233	-.147	.245	-.002	.367*	.119	.135	-.214	-.185	-.077	.286	-.269	.153	-.293	.077	.182	-.046
	p	.619	.119	.131	.377	.580	.865	.796	.172	.393	.151	.993	.028	.639	.593	.395	.463	.768	.266	.297	.557	.223	.753	.455	.853
miR-181a	r	.040	-.335	.163	.269	.331*	-.214	-.144	.107	.003	.115	-.105	.369*	.067	-.185	.125	-.286	-.102	.313	-.228	.112	-.332	.123	.168	.082
	p	.820	.053	.358	.124	.049	.211	.401	.536	.986	.505	.541	.027	.791	.463	.622	.250	.687	.206	.363	.660	.166	.616	.491	.737
miR-222	r	.115	-.418*	.220	.259	.277	-.207	-.079	.012	.159	.007	-.267	.340*	-.022	.064	-.017	-.368	-.152	.389	-.222	.078	-.292	.098	.168	-.183
	p	.516	.014	.211	.139	.107	.234	.651	.947	.354	.966	.115	.042	.933	.808	.948	.147	.548	.111	.376	.757	.240	.699	.505	.468
miR-103	r	.012	-.340*	.248	.161	.348*	-.331*	.029	.136	.111	-.030	-.125	.406*	.191	-.271	.053	-.195	-.166	.486*	-.267	.032	-.368	.166	.200	.039
	p	.945	.049	.158	.364	.037	.048	.866	.430	.520	.860	.466	.014	.448	.276	.836	.438	.510	.041	.284	.900	.133	.510	.425	.877
miR-30a	r	.061	-.315	.162	.220	.316	-.197	-.082	.190	-.034	.129	-.092	.448**	.199	-.100	-.224	-.416	.034	.195	-.309	.118	-.404	.247	.119	-.033
	p	.735	.074	.366	.219	.064	.258	.640	.275	.843	.454	.592	.006	.428	.693	.372	.086	.893	.438	.213	.642	.087	.307	.627	.892
miR-27a	r	-.003	-.252	.141	.265	-.011	.172	-.098	.296	-.094	.128	.038	.272	.001	.094	-.181	-.422	.065	.356	-.519*	.249	-.376	.336	-.086	-.001
	p	.986	.151	.427	.131	.950	.316	.568	.080	.584	.455	.827	.108	.997	.711	.473	.081	.798	.147	.027	.319	.112	.159	.726	.997
miR-27b	r	.144	-.339*	.114	.264	.114	.036	-.122	.054	-.033	.135	-.083	.188	.053	.121	-.156	-.356	-.011	.350	-.389	.125	-.277	.267	-.169	-.064
	p	.416	.050	.520	.132	.506	.834	.479	.756	.851	.440	.634	.281	.836	.633	.537	.147	.964	.155	.111	.621	.282	.300	.516	.808
miR-200b	r	.040	-.401*	.268	.232	.171	-.142	-.123	-.075	.149	.020	-.290	.383*	.057	-.150	.160	-.368	-.380	.512*	.039	-.075	-.453	.123	.323	-.163
	p	.824	.019	.125	.188	.350	.438	.503	.684	.401	.909	.096	.025	.823	.553	.526	.132	.133	.036	.881	.775	.052	.616	.178	.505
miR-let7a	r	.115	-.247	-.008	.428*	.503**	-.352	-.294	-.006	-.101	.245	-.033	.178	.125	-.091	-.027	-.449	.025	.164	-.517*	.130	-.321	.067	.183	-.079
	p	.518	.158	.965	.012	.004	.052	.109	.974	.575	.169	.856	.320	.633	.729	.918	.071	.926	.529	.034	.619	.194	.791	.468	.754
miR-17	r	.102	-.373*	.135	.231	.434*	-.284	-.183	.185	-.041	.170	-.090	.445**	.117	-.148	-.069	-.385	.036	.162	-.321	.123	-.342	.098	.193	.018
	p	.565	.030	.448	.189	.010	.104	.299	.295	.813	.323	.602	.006	.645	.559	.785	.115	.887	.521	.194	.627	.152	.689	.429	.943

miR-let7b	r	.105	-.342*	.129	.270	.450**	-.304	-.168	.161	.006	.142	-.137	.425**	.044	-.187	.123	-.383	.024	.199	-.360	.185	-.260	-.004	.256	-.040
	p	.554	.047	.467	.122	.006	.072	.328	.349	.971	.408	.427	.010	.861	.458	.627	.117	.926	.428	.142	.463	.283	.989	.290	.870
miR-let7c	r	.150	-.274	.058	.197	.414*	-.295	-.160	.136	.143	.058	-.302	.465**	.020	-.102	.092	-.321	.238	.150	-.602**	.151	-.211	-.051	.256	-.026
	p	.403	.122	.748	.272	.012	.081	.351	.430	.405	.736	.074	.004	.938	.687	.717	.194	.341	.553	.008	.550	.387	.836	.290	.915
miR-146b	r	-.075	-.207	.202	.196	.275	-.094	-.176	.033	.090	.083	-.142	.450**	.143	-.234	.009	-.453	-.203	.387	-.162	.001	-.246	.009	.212	.012
	p	.683	.256	.268	.282	.134	.614	.344	.862	.612	.641	.423	.008	.570	.349	.971	.059	.434	.125	.535	.996	.311	.972	.383	.960

Whole population was segregated at sampling time in normal-weight mothers (BMI<25) and overweight/obese (BMI≥25). Associations between miRNA expression and breast milk lipid composition were tested by Spearman's correlation test at three months. Spearman's rank correlation coefficient (r) and significance (p) is shown.

** = p< 0.01 (bilateral); * = p< 0.05 (bilateral)
